# Minimal Fat Content in Papillary Renal Cell Carcinoma Diagnosed with Dual-Layer Dual-Energy CT

**DOI:** 10.3390/diagnostics13101742

**Published:** 2023-05-15

**Authors:** Velio Ascenti, Francesco M. Arico, Renato Trimarchi, Giuseppe Cicero, Antonio Ieni, Marta Rossanese, Giorgio Ascenti

**Affiliations:** 1 Postgraduate School of Radiodiagnostics, Policlinico Universitario, University of Milan, 20133 Milano, Italy; velio.ascenti@unimi.it; 2 Diagnostic and Interventional Radiology Unit, BIOMORF Department, University Hospital “Policlinico G. Martino”, 98124 Messina, Italy; renatotrimarchi@live.it (R.T.); giuseppe.cicero@unime.it (G.C.); giorgio.ascenti@unime.it (G.A.); 3 Department of Human Pathology of Adult and Evolutive Age “Gaetano Barresi”-Section of Pathological Anatomy, University of Messina, Viale Gazzi, 98125 Messina, Italy; antonio.ieni@unime.it; 4 Gaetano Barresi Department of Human and Paediatric Pathology, Urologic Section, University of Messina, 98166 Messina, Italy; marta.rossanese@unime.it

**Keywords:** papillary renal cell carcinoma, angiomyolipoma, dual-energy CT, spectral CT, computed tomography, spiral, diagnostic techniques, fat imaging, kidney neoplasms

## Abstract

A 56-year-old man with a previous right nephrectomy for multiple papillary renal cell carcinomas (pRCC) underwent a follow-up CT scan. Using a dual-layer dual-energy CT (dlDECT), we demonstrated the presence of a small amount of fat in a 2.5 cm pRCC that mimicked the diagnosis of angiomyolipoma (AML). Histological examination demonstrated the absence of macroscopic intratumoral adipose tissue, showing a fair amount of enlarged foam macrophages loaded with intracytoplasmic lipids. The presence of fat density in an RCC is an extremely rare occurrence in the literature. To our knowledge, this is the first description using dlDECT of a minimal amount of fat tissue in a small RCC due to the presence of tumor-associated foam macrophages. Radiologists should be aware of this possibility when characterizing a renal mass with DECT. The option of RCCs must be considered, especially in the case of masses with an aggressive character or a positive history of RCC.

The detection of fat-density components represents a key diagnostic criterion for the computed tomography (CT) characterization of a renal mass (Figure 1A,B). The presence of macroscopic adipose tissue in the context of a solid, vascularized renal mass without calcifications is widely considered a reliable feature for CT characterization of renal angiomyolipoma (AML). This diagnosis is to be obtained through the measurement of negative Hounsfield numbers in a thin-slice, unenhanced CT scan. On the contrary, adipose densities associated with calcifications are expressions of osseous metaplasia, constituting a specific, although rare, finding of renal cell carcinoma (RCC) because calcifications within AML have not been reported in the radiologic literature. An insight into the main differential diagnoses in fat-containing renal masses has been accurately described by Helenon et al. [1]. However, with the advent of dual-energy CT (DECT), other analysis systems such as iodine maps (Figure 2), virtual monoenergetic imaging with spectral curves (Figure 3A,B), atomic-number maps (Figure 4A) and fat maps (Figure 4B) enhance the signal fat and increase the sensitivity of CT in the recognition of intralesional adipose tissue. 

We describe the case of a 56-year-old man with a history of right nephrectomy due to papillary renal cell carcinoma who underwent a follow-up CT scan. Using a dual-layer DECT (dlDECT) (IQon Spectral CT, Philips Healthcare, Best, The Netherlands), we confirmed the presence of a small amount of fat in a 2.5 cm papillary renal cell carcinoma (pRCC) that mimicked the diagnosis of AML. Histological examination revealed a significant number of foam macrophages and the absence of macroscopic intratumoral adipose tissue (Figure 5).

Histological examination demonstrated the absence of macroscopic intratumoral adipose tissue, showing a fair amount of foam macrophages. As with other solid tumors, RCCs comprise a heterogeneous microenvironment of both malignant and normal stromal cells that contain a large number of macrophages, originating from blood monocytes attracted by chemokines and cytokines produced by tumor cells. Tumor-associated macrophages were demonstrated to directly stimulate tumor cell proliferation and angiogenesis [3]. They are considered a potential biomarker of aggressive behavior and are related to a poor prognosis [4]. These macrophages became enlarged and loaded with lipids such as cholesterol in the cytoplasm, adopting a foamy appearance upon microscopic examination that can be present in up to 30% of renal masses and that represents a histological hallmark of papillary RCC [5]. The presence of fat density in an RCC is an extremely rare occurrence in the literature [6,7,8]. Besides the well-known coexistence of fat and calcifications due to osseous metaplasia, two other mechanisms are reported in the literature for large RCCs: lipid vacuoles and amalgamated cholesterol clefts due to necrosis and entrapment of perirenal or sinus fat [1,5]. MRI using fat suppression techniques can provide cues for the detection of macroscopic fat tissue in the case of a renal tumor. Currently, MRI with in-phase and out-of-phase sequences is the most effective imaging technique for evaluating intracytoplasmatic, tumoral, and microscopic fat [9]. Dual-energy CT (DECT) has recently been shown to be capable of detecting intratumoral fat, and doing so may aid in differentiating clear-cell RCC from other enhancing renal tumors. They achieved this by using material-specific fat images from dual-energy rapid-kVp-switch CT [10]. To the best of our knowledge, our case is the first example of using dlDECT to show that fat attenuation within a hypovascular renal mass can result from the presence of foam macrophages, a histological hallmark of papillary RCC, rather than extracellular macroscopic fat. In our opinion, the occurrence of fat density due to the presence of foam macrophages should be included among other known causes of adipose attenuation in RCC, when a DECT is used. Radiologists should be aware of this possibility when defining a renal mass using DECT. 

## Figures and Tables

**Figure 1:**
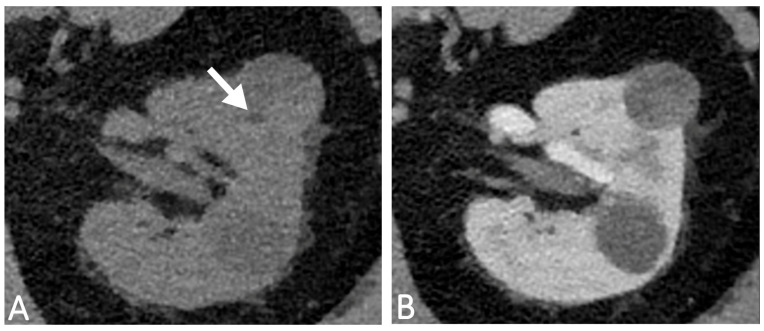
56-year-old man with a history of right nephrectomy due to multiple pRCC. Follow-up CT scans obtained before (**A**) and after contrast injection (**B**) show a 2.5 cm heterogeneous mass within the left kidney, with small hypodense focus not clearly related to fat (white arrow) and very subtle contrast enhancement. No calcifications within the mass were identified.

**Figure 2:**
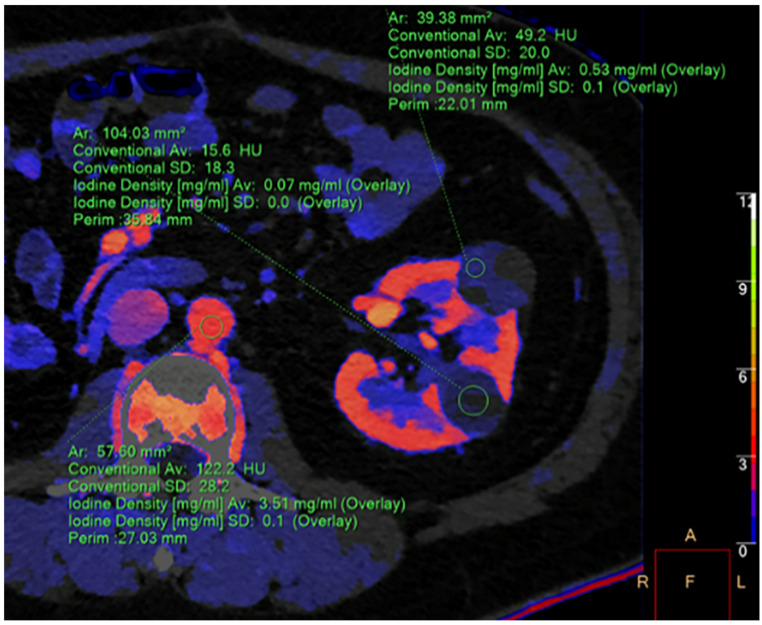
In the contrast-enhanced iodine map CT, iodine-containing pixels are assigned values equal to the concentration of iodine, expressed in mg/mL [2]. The image shows a 2.5 cm inhomogeneous hypovascular anterior renal mass (iodine concentration 0.57 mg/mL) consistent with papillary renal cell carcinoma and a 2.2 cm avascular posterior renal mass (iodine concentration 0.07 mg/mL) representing a simple cyst. A third region of interest in the abdominal aorta shows an iodine concentration of 3.5 mg/mL.

**Figure 3:**
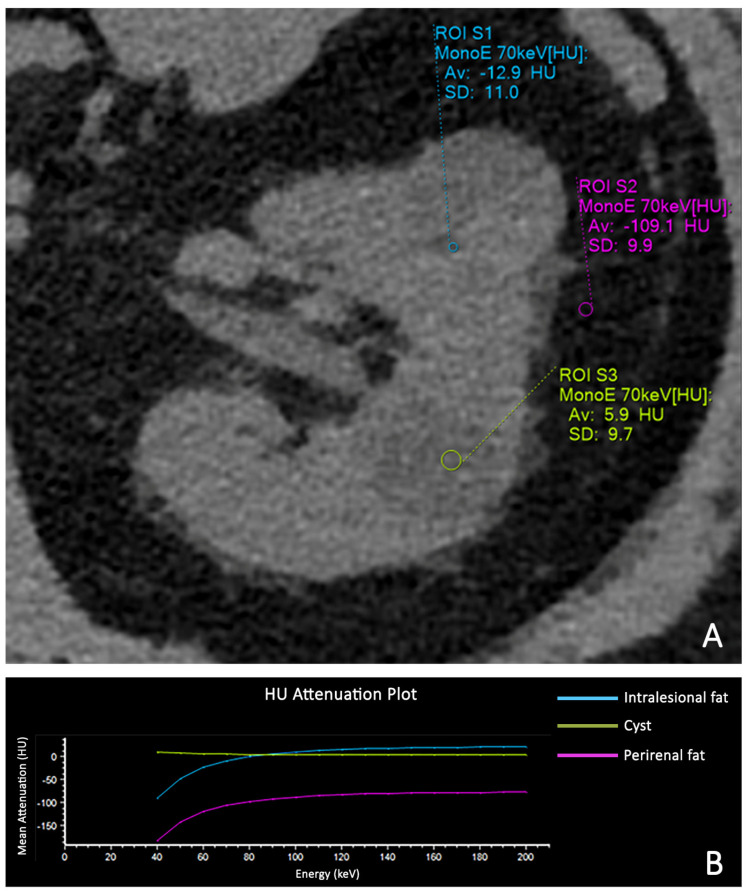
Unenhanced monochromatic 70 keV CT image showing a minimal amount of hypodense tissue in the rear part of the anterior mass with negative values (Hounsfield Unit −12.9). The other two ROIs are placed in the cyst (HU 5.9) and in the perirenal fat (HU −109.1) (**A**). HU attenuation curves derived from unenhanced monochromatic CT images from 40 to 220 keV. Both intratumoral hypodense foci (blue line) and perirenal fat (purple line) exhibit a decrease in attenuation as energy is reduced, which is characteristic of adipose tissue. No difference in curve attenuation is visible for a cystic mass (green line) (**B**).

**Figure 4:**
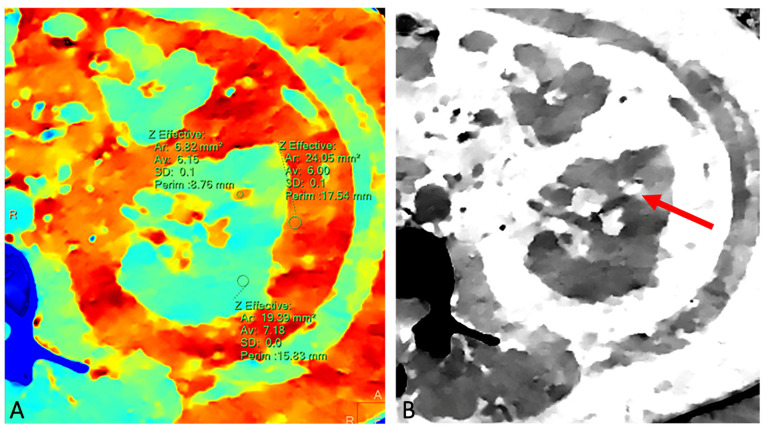
Unenhanced z-effective map CT image demonstrating that the atomic number of small intralesional hypodensity (6.15) is very close to that of perirenal fat (6.0) (**A**). Unenhanced fat map CT image showing that the attenuation of the small intralesional component (red arrow) increases, as does that of the perirenal and intra-abdominal fat (**B**).

**Figure 5:**
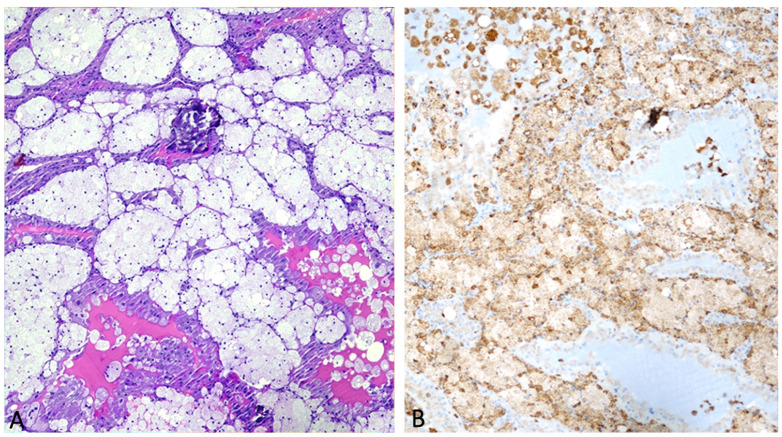
At low magnification, the characteristic growth pattern of papillary carcinoma with well-formed papillary structures containing variable amounts of foamy macrophages is represented (hematoxylin and eosin, 20×) (**A**). The accumulation of macrophages stains positive exclusively for CD68 (nuclear Mayer’s hemalum counterstain, 20×) (**B**).

## Data Availability

Data are contained within the article.
